# Supplementary material for: Sequential i-GONAD: An Improved In Vivo Technique for CRISPR/Cas9-Based Genetic Manipulations in Mice
Source: Cells. 2020 Feb 26;9(3):546. doi: 10.3390/cells9030546 (PMC7140409; doi:10.3390/cells9030546)
Supplement: Supplementary file 1 [file cells-09-00546-s001.pdf]

Suppl. Table 1. Detailed results of *si*-GONAD, *simi*-GONAD and *i*-GONAD targeted to exon 4 of murine *GGTA1*.

Method[1]	Sample name of single embryos	Pattern of mutations in A and B sites of exon 4 of *GGTA1*[2]	Sequence at exon 4 of mouse *GGTA1*[3]	Levels of FITC-dextran-derived fluorescence[4]	Levels of fluorescence after staining with AF594-IB4[5]
si-GONAD	#1L-1	(- : B)	AATAATGAATGTCAAGGGAAAAGTAATCCTGTTGATGCTGATTGTCTCAAC<br>CGTGGTTGTCGTGTTTTGGGAATATGTCACCAGGTAATTAT	++	++
si-GONAD	#1L-2	(A: -)	AATAATGAATGGCAAGGGAAAAGTAATCCTGGTGATGCTGATTGGCTCAAC<br>CGTGGTTGTCGTGTTTTGGGAATATGTCAACAGGTAATTAT	+	++
si-GONAD	#1L-3	Complex	AANNANGNANNGNCANGGNAAAATNANNCNGGTNNANNCNNATGGNCN<br>CACCCGGGNTTGTNNGGTTTTGGAANTTTTTCA	+	++
si-GONAD	#1L-4	(- : -)	AATAATGAATGTCAAGGGAAAAGTAATCCTGTTGATGCTGATTGTCTCAAC<br>CGTGGTTGTCGTGTTTTGGGAATATGTCAACAGGTAATTAT	++	++
si-GONAD	#1L-5	Complex	AAANANNAATNNNNANGGNAAAANNANCCNGGTGGNGGCGNNTGGNNC<br>CANCGGGGNTGGNNNGGTTTNGNAANNNTGTCAAAGGGG	+	++
si-GONAD	#1L-6	(- : B)	AATAATGAATGTCAAGGGAAAAGTAATCCTGTTGATGCTGATTGTCTCAAC<br>CGTGGTTGTCGTGTTTTGGGAATAGAAAGGCTGCTTT	++	++
si-GONAD	#1L-7	(- : B)	AATAATGAATGTCAAGGGAAAAGTAATCCTGTTGATGCTGATTGTCTCAAC<br>CGTGGTTGTCGTGTTTTGGGAACAGGTCATTAT	+	+
si-GONAD	#1L-8	(- : B)	AATAATGAATGTCAAGGGAAAAGTAATCCTGTTGATGCTGATTGTCTCAAC<br>CGTGGTTGTCGTGTTTTGGGAAAGCTAGAAAGGCTGCTTT	±	++
si-GONAD	#1L-9	(- : -)	AATAATGAATGTCAAGGGAAAAGTAATCCTGTTGATGCTGATTGTCTCAAC<br>CGTGGTTGTCGTGTTTTGGGAATATGTCAACAGGTAATTAT	±	±
si-GONAD	#1L-10	(A: B)	AATGAATGGCAAGGGAAAAGTAATCCTGGTGATGCTGATTGGCTCAACCG<br>TGGTTGGCGTGGTTTGGGAACAGGGAATTAT	++	±
si-GONAD	#1L-11	(- : B)	AATAATGAATGTCAAGGGAAAAGTAATCCTGTTGATGCTGATTGTCTCAAC<br>CGTGGTTGTCGTGTTATGGGTAATTAT	+	±
si-GONAD	#2R-3	(- : B)	AATAATGAATGTCAAGGGAAAAGTAATCCTGTTGATGCTGATTGTCTCAAC<br>CGTGGTTGTCGTGTTTTGGGAATTATGAAGCCAGC	+	++
si-GONAD	#2R-4	(A: B)	AATAATGAATGTTCAAGGGAAAAGTAATCCTGTTGATGCTGATTGTCTCAA<br>CCGTGGTTGTCGTGTTTTGGGAATATGTAACAGGTAATTAT	+	++
si-GONAD	#2R-5	(- : B)	AATAATGAATGTCAAGGGAAAAGTAATCCTGTTGATGCTGATTGTCTCAAC<br>CGTGGTTGTGGTGTTTTGGGAATNNTCCNGN	+	++
si-GONAD	#2R-6	(- : B)	AATAATGAATGTCAAGGGAAAAGTAATCCTGTTGATGCTGATTGTCTCAAC<br>CGTGGTTGTCGTGTTTTGGGAATATGTCAGGTAATTAT	+	++
si-GONAD	#2R-7	(A: B)	AATAATGAAGGTCAGGGGAAAAGTAATCCGGTGGATGCTGATGGGCTCAC<br>CCGGGGTTNGA	+	±
si-GONAD	#2R-8	(- : B)	AATAATGAATGTCAAGGGAAAAGTAATCCTGTTGATGCTGATTGTCTCAAC<br>CGTGGTTGTCGTGTTTTGGTAATTAT	+	±
si-GONAD	#2R-9	(A : B) (mosaic?)	AANNANNNAANGNNNNGGNAAAANTAATCCTGTTNANGCTNATTGTCTCA<br>NCCNTGGTTGTCNGGTTTTGGNAATATGTCAACAGGTAATTAT	++	++
si-GONAD	#2R-10	(A : B)	AATAATGAAAAGTAAGGGAAAAGTAATCCTGTTGATGCTGATTGTCTCAAC<br>CGTGGTTGTCGTGTTTTGGGAATATGTCTAACAGGTAATTAT	+	++
si-GONAD	#2R-11	Complex (LD?)	AAANGNANGGNAANGGAAAAANAAACCCGGTTAAGGCNAATGGCCCNAN<br>CCGGGNTGNCCTGGTTTGGGNANANNNTCGTTATTAAGGAACCAGCCAGG<br>AAGGC	±	++
si-GONAD	#2R-12	(- : B)	AATAATGAATGTCAAGGGAAAAGTAATCCTGTTGATGCTGATTGTCTCAAC<br>CGTGGTTGTCGTGTTTTGGGAATATGGCTGCTTTCA	-	++
si-GONAD	#2R-13	LD	AAGCCATCTCTCATTCCAGATTTAGTTTATTTAATCTATTTCCCCCTCTTATTT<br>CTCCCTGCCTCTACAGGAGAAAATAATGAATGTCAAGGGAAAAGTAATCCT<br>GTTGATGCTGATTGTCTCAACCGTGGTTGTCGTGTTTCATTCCCTGTGACTG	-	++

si-GONAD	#3R-1	Complex	AATAATNANNGNTCAGGGAAAANNNNNCCNNNNNNNNGNNNANNGTCNC<br>NCCCGGGTTTGCCGGGTTTNGGAAATNTNTCANCGGGAATT	+	++
si-GONAD	#3R-2	(- : -)	AATAATGAATGTCAAGGGAAAAGTAATCCTGTTGATGCTGATTGTCTCAAC<br>CGTGGTTGTCGTGTTTTGGGAATATGTCAACAGGTAATTAT	+	++
si-GONAD	#3R-3	(- : -)	AATAATGAATGTCAAGGGAAAAGTAATCCTGTTGATGCTGATTGTCTCAAC<br>CGTGGTTGTCGTGTTTTGGGAATATGTCAACAGGTAATTAT	+	++
si-GONAD	#3R-4	(- : -)	AATAATGAATGTCAAGGGAAAAGTAATCCTGTTGATGCTGATTGTCTCAAC<br>CGTGGTTGTCGTGTTTTGGGAATATGTCAACAGGTAATTAT	+	±
si-GONAD	#3R-5	(- : -)	AATAATGAATGTCAAGGGAAAAGTAATCCTGTTGATGCTGATTGTCTCAAC<br>CGTGGTTGTCGTGTTTTGGGAATATGTCAACAGGTAATTAT	+	±
si-GONAD	#3R-6	(- : -)	AATAATGAATGTCAAGGGAAAAGTAATCCTGTTGATGCTGATTGTCTCAAC<br>CGTGGTTGTCGTGTTTTGGGAATATGTCAACAGGTAATTAT	-	++

simi-GONAD	#1R-1	Complex	AANNANGNATGNNNAGGGNAAAATAATCCTGGTGAAGCTGAATGGCTCA<br>ACCGNGGNTNNNNNGGTTTGGGAANNTNTTNANCGGTTATTTTNAAACCC	++	±
simi-GONAD	#1R-2	(- : B)	AATAATGAATGTCAAGGGAAAAGTAATCCTGTTGATGCTGATTGTCTCAAC<br>CGTGGTTGTCGTGTTTTGGGAAAATTACAACGGCTGCTTTC	++	++
simi-GONAD	#1R-3	(- : -)	AATAATGAATGTCAAGGGAAAAGTAATCCTGTTGATGCTGATTGTCTCAAC<br>CGTGGTTGTCGTGTTTTGGGAATATGTCAACAGGTAATTAT	++	++
simi-GONAD	#1R-4	(- : B)	AATAATGAATGTCAAGGGAAAAGTAATCCTGGTGATGCTGATTGGCTCAAC<br>CGTGGNTGGCNNGGTTTGGGAATATGTCAACANGGNANTNNNAANCCCNC	++	++
simi-GONAD	#1R-5	(- : -)	AATAATGAATGTCAAGGGAAAAGTAATCCTGTTGATGCTGATTGTCTCAAC<br>CGTGGTTGTCGTGTTTTGGGAATATGTCAACAGGTAATTAT	++	++
simi-GONAD	#1R-6	(- : -)	AATAATGAATGTCAAGGGAAAAGTAATCCTGTTGATGCTGATTGTCTCAAC<br>CGTGGTTGTCGTGTTTTGGGAATATGTCAACAGGTAATTAT	+	++
simi-GONAD	#1R-7	(- : -)	AATAATGAATGTCAAGGGAAAAGTAATCCTGTTGATGCTGATTGTCTCAAC<br>CGTGGTTGTCGTGTTTTGGGAATATGTCAACAGGTAATTAT	+	++
simi-GONAD	#1R-8	(- : -)	AATAATGAATGTCAAGGGAAAAGTAATCCTGTTGATGCTGATTGTCTCAAC<br>CGTGGTTGTCGTGTTTTGGGAATATGTCAACAGGTAATTAT	+	++
simi-GONAD	#1R-9	Complex	GNNAAAANNANNNANGGNNNNGGNAAAANNATCCTGGTNANGCTGAATG<br>GCCCCACCCGGGNTGNCCNGGTTTNGGNANNNNTNCACCNGGNATTTTTA	++	±
simi-GONAD	#1R-10	(- : -)	AATAATGAATGTCAAGGGAAAAGTAATCCTGTTGATGCTGATTGTCTCAAC<br>CGTGGTTGTCGTGTTTTGGGAATATGTCAACAGGTAATTAT	+	++
simi-GONAD	#1R-11	(- : -)	AATAATGAATGTCAAGGGAAAAGTAATCCTGTTGATGCTGATTGTCTCAAC<br>CGTGGTTGTCGTGTTTTGGGAATATGTCAACAGGTAATTAT	+	++
simi-GONAD	#1R-12	(- : -)	AATAATGAATGTCAAGGGAAAAGTAATCCTGTTGATGCTGATTGTCTCAAC<br>CGTGGTTGTCGTGTTTTGGGAATATGTCAACAGGTAATTAT	±	++
simi-GONAD	#1L-1	(- : -)	AATAATGAATGTCAAGGGAAAAGTAATCCTGTTGATGCTGATTGTCTCAAC<br>CGTGGTTGTCGTGTTTTGGGAATATGTCAACAGGTAATTAT	++	++
simi-GONAD	#1L-2	(- : -)	AATAATGAATGTCAAGGGAAAAGTAATCCTGTTGATGCTGATTGTCTCAAC<br>CGTGGTTGTCGTGTTTTGGGAATATGTCAACAGGTAATTAT	+	±
simi-GONAD	#1L-3	(- : -)	AATAATGAATGTCAAGGGAAAAGTAATCCTGTTGATGCTGATTGTCTCAAC<br>CGTGGTTGTCGTGTTTTGGGAATATGTCAACAGGTAATTAT	+	++
simi-GONAD	#1L-4	(- : -)	AATAATGAATGTCAAGGGAAAAGTAATCCTGTTGATGCTGATTGTCTCAAC<br>CGTGGTTGTCGTGTTTTGGGAATATGTCAACAGGTAATTAT	+	++
simi-GONAD	#1L-5	(- : -)	AATAATGAATGTCAAGGGAAAAGTAATCCTGTTGATGCTGATTGTCTCAAC<br>CGTGGTTGTCGTGTTTTGGGAATATGTCAACAGGTAATTAT	+	±
simi-GONAD	#1L-6	(- : -)	AATAATGAATGTCAAGGGAAAAGTAATCCTGTTGATGCTGATTGTCTCAAC<br>CGTGGTTGTCGTGTTTTGGGAATATGTCAACAGGTAATTAT	+	++
simi-GONAD	#1L-7	Complex	AATAANGAANGNNNAGGGAAAAATAATCCTGGTGATGCTGATTGTCTCNA<br>CCGNGGTTGTCCTGTTTTGGGAATATGTCAACNNGTAATTAT	±	++
simi-GONAD	#1L-8	(- : -)	AATAATGAATGTCAAGGGAAAAGTAATCCTGTTGATGCTGATTGTCTCAAC<br>CGTGGTTGTCGTGTTTTGGGAATATGTCAACAGGTAATTAT	±	++

simi-GONAD	#1L-9	Complex	AATAATGAATGNNNAGGGAAAAGTAATCCTGGTGATGCTGATTGGCTCAACCCNGGNTGGCCNGGTTTGGGAATATTTCNACCNGGAATTATTAAACCCG	±	++
simi-GONAD	#1L-10	(- : -)	AATAATGAATGTCAAGGGAAAAGTAATCCTGTTGATGCTGATTGTCTCAACCGTGGTTGTCGTGTTTTGGGAATATGTCAACAGGTAATTAT	-	++
i-GONAD	#1R-1	(- : B)	AATAATGAATGTCAAGGGAAAAGTAATCCTGTTGATGCTGATTGTCTCAACCGTGGTTGTCGTGTTTTGGGAATTATGAAGC	±	++
i-GONAD	#1R-2	Complex	AANNNANGGNNANGGAAAANTTATCCTGGTTAAGGCTANTGNNCCNACCCNGGNTTGCTTGGTTTNCNNNNAAAAGGGNCCTTTTAATT	±	++
i-GONAD	#1R-3	(- : B)	AATAATGAATGTCAAGGGAAAAGTAATCCTGTTGATGCTGATTGTCTCAACCGTGGTTGTCGTGTTTTGGGAATATAACAGGTAATTAT	±	++
i-GONAD	#1R-4	(- : B)	AATAATGAATGTCAAGGGAAAAGTAATCCTGTTGATGCTGATTGTCTCAACCGTGGTTGTCGTGTTTGGGGAGATATNAAANNCGGNTGGA	±	++
i-GONAD	#1R-5	LD	CTGCTTTCATTCCCTGTGACTGGTGCCAGCTGAGTGACCAATCAGTCTGAACATAAGGGACGGAGCCGTGAGCAGGAGTCCAGTCTTCCTGTGTTCC	±	++
i-GONAD	#1R-6	(- : B)	AATAATGAATGTCAAGGGAAAAGTAATCCTGTTGATGCTGATTGTCTCAACCGTGGTTGTCGTGTTTTGGGAATAGAAAANGNAATNTTNAT	±	++
i-GONAD	#1R-7	(- : B)	AATAATGAATGGCAAGGGAAAAGTTATCCTGGTGAAGNTTACTTTTCCCTANNCGGTNNTNATTTTNNNNGGNAAGGGNGNAGAAAAANCAGGNNGAAAGGC	+	±
i-GONAD	#1R-8	(- : B)	AATAATGAATGTCAAGGGAAAAGTAATCCTGTTGATGCTGATTGTCTCAACCGTGNNCGTTGTGTTTCTTATTTTNNCCNGNNANGGAAGANTNAAAAACA	+	±
i-GONAD	#1R-9	(- : B)	AATAATGAATGTCAAGGGAAAAGTAATCCTGTTGATGCTGATTGTCTCAACCGTGGTTGTCGTGTTTTGGGAACTGGGAATTATGAANCCAGNTANGATGGCTGTTTTCAT	±	++
i-GONAD	#1R-10	(- : B)	AATAATGAATGTCAAGGGAAAAGTAATCCTGTTGATGCTGATTGTCTCAACCGTGGTTGTCGTGTTTTGGGAATATGTCTAACAGGTAATTTT	±	++

[1]*si*-GONAD was performed by the 1st *i*-GONAD with a solution containing Cas9/#6 gRNA complex and FITC-dextran and subsequently the 2nd *i*-GONAD with a solution containing Cas9/Ex4 gRNA complex. *simi*-GONAD was performed by *i*-GONAD with a solution containing Cas9/#6 gRNA complex, FITC-dextran and Cas9/Ex4 gRNA complex. *i*-GONAD was performed using a solution containing Cas9/#6 gRNA complex and FITC-dextran.
[2]Mutations (indels) occurred on the A or B site of exon 4 of *GGTA1* are defined as (A : -) and (- : B), respectively. Mutations induced on both sites are defined as (A : B). “Complex” means that several mutations are mixed in one embryo. LD indicates large deletion in a region spanning exon 4 of *GGTA1*. No mutation on both sites seen in an embryo is defined as (- : -).
[3]Sequences of exon 4 of *GGTA1* which includes ATG site are shown.
[4]Intensity of fluorescence in each embryo after observation under a fluorescence microscope is determined as – (no fluorescence), ± (very fain fluorescence), + (moderate fluorescence), and ++ (strong fluorescence).
[5]Intensity of fluorescence in each embryo after staining with AF594-IB4 is determined as – (no fluorescence), ± (very fain fluorescence), + (moderate fluorescence), and ++ (strong fluorescence).

Suppl. Table 2. Detailed results of *si*-GONAD- and *simi*-GONAD-based KI targeted to exon 4 of murine *Mecp2*.

Method[1]	Sample name of single embryos[2]	Site of intron to be knocked-in[3]	Mode of KI or indels[4]	Sequence at 5' or 3' intronic region flanking exon 4 of mouse *Mecp2*[5]	Levels of FITC-dextran-derived fluorescence[6]
si-GONAD	#1R-1	5' intron	Intact	TTTGGGCCCCAGCTTGACCCAAGGATACAGTATCCTAGGGAAGTTACCAAAATCAGAGA<br>TAGTATGCAGCAGCCAGGGGTCTCATGTGT	++
		3' intron	Indels	AAACTTNNTANNTTNGTATAAGGTTNATTCCCGCACGTTAATTGGCCTCAATCCTTCAGT<br>TTAAGGATTGTGGAAAAGCCAGGG	
si-GONAD	#1R-2	5' intron	Indels	TTTGGGCCCCAACTTGACCCAAGGATACAGTATCCTAGGGAAGTTACCAAAATCAGAGA<br>TAGTATGCAGCAGCCAGGGGTCTCATGTGT	+
		3' intron	Intact	AAAGATATTCAGTCTAGCCCAATGACCCCCAAGTACTAGACCTCACTCCTTCAGTTTAA<br>GGATTGTGGAAAA	
si-GONAD	#1R-3	5' intron	Intact	TTTGGGCCCCAGCTTGACCCAAGGATACAGTATCCTAGGGAAGTTACCAAAATCAGAGA<br>TAGTATGCAGCAGCCAGGGGTCTCATGTGT	±
		3' intron	Intact	AAAGATATTCAGTCTAGCCCAATGACCCCCAAGTACTAGACCTCACTCCTTCAGTTTAA<br>GGATTGTGGAAAA	
si-GONAD	#1R-4	5' intron	Intact	TTTGGGCCCCAGCTTGACCCAAGGATACAGTATCCTAGGGAAGTTACCAAAATCAGAGA<br>TAGTATGCAGCAGCCAGGGGTCTCATGTGT	++
		3' intron	Intact	AAAGATATTCAGTCTAGCCCAATGACCCCCAAGTACTAGACCTCACTCCTTCAGTTTAA<br>GGATTGTGGAAAA	
si-GONAD	#1R-5	5' intron	Intact	TTTGGGCCCCAGCTTGACCCAAGGATACAGTATCCTAGGGAAGTTACCAAAATCAGAG<br>ATAGTATGCAGCAGCCAGGGGTCTCATGT	+
		3' intron	Intact	AAAGATATTCAGTCTAGCCCAATGACCCCCAAGTACTAGACCTCACTCCTTCAGTTTA<br>AGGATTGTGGAAAA	
si-GONAD	#1R-6	5' intron	Intact	TTTGGGCCCCAGCTTGACCCAAGGATACAGTATCCTAGGGAAGTTACCAAAATCAGAG<br>ATAGTATGCAGCAGCCAGGGGTCTCATGT	++
		3' intron	Intact	AAAGATATTCAGTCTAGCCCAATGACCCCCAAGTACTAGACCTCACTCCTTCAGTTTA<br>AGGATTGTGGAAAA	
si-GONAD	#1R-7	5' intron	Intact	TTTGGGCCCCAGCTTGACCCAAGGATACAGTATCCTAGGGAAGTTACCAAAATCAGAG<br>ATAGTATGCAGCAGCCAGGGGTCTCATGT	±
		3' intron	Intact	AAAGATATTCAGTCTAGCCCAATGACCCCCAAGTACTAGACCTCACTCCTTCAGTTTA<br>AGGATTGTGGAAAA	
si-GONAD	#1R-8	5' intron	Indels	TTTGGGGCCCTTGTTGGCCCGNGATACNGTATCTTTCAAATAACTCTGTTTTGGCTTCT<br>AAANAGCAGCCAGGGGGGTCATGTGTGGGGGT	±
		3' intron	Indels	ACAGATATTCAGTCTAGCCCAAAGACCCCCAAGTANTAGACCTCACTCNNNCANNTC<br>AAGGACTGAGGAAAA	
si-GONAD	#1R-9	5' intron	Intact	TTTGGGGCCCTTGTTGGCCCGNGATACNGTATCTTTCAAATAACTCTGTTTTGGCTTCT<br>AAANAGCAGCCAGGGGGGTCATGTGTGGGGGT	-
		3' intron	Intact	AAAGATATTCAGTCTAGCCCAATGACCCCCAAGTACTAGACCTCACTCCTTCAGTTTA<br>AGGATTGTGGAAAA	
si-GONAD	#1R-10	5' intron	Intact	TTTGGGCCCCAGCTTGACCCAAGGATACAGTATCCTAGGGAAGTTACCAAAATCAGAG<br>ATAGTATGCAGCAGCCAGGGGTCTCATGT	-
		3' intron	Indels	AAAGATATTCAGTCCAGCCCAATCACCCCCAAGTACTAGACCTCACTCCNNTCANNNT<br>NNNNTGNTNTNNNNANNACG	
si-GONAD	#1L-1	5' intron	KI	TTTGGGCCCCAGCTTGACCCAAGGATACAGTATCCCTAGCTACCGTTCGTATAATGTAT<br>GCTATACGAAGGTTATCCTAGGGAAGTTACCAAAATCAGAGATAGTATGCAGCAGCCA<br>GGGGTC	++
		3' intron	Indels	AAAGATATTCAGTCCCNNCCANNNACCCCCAAGTACTACACCTCACTNCATCAGNNTA<br>NCNGANNTNNGNCATNNNNNCACAGGGACNACNAGTGGCAGNNNGGNNNNNN	

si-GONAD	#1L-2	5’ intron	ND		++
		3’ intron	ND		
si-GONAD	#1L-3	5’ intron	Indels	TTTGGGCCCCAGCTTGNCCCAACGATACAGTATACATATACANNNGGAAGTTACCATG ATCTCAGATAGTATGCAGCTGCCTTGGGACTCATGTGTGGCACTC	++
		3’ intron	Indels	AACNNNAGATATNCAGTCTCGCCCAATGGCCCCCAAGTACTAGACCTCACTCCTTCAG TTTAAGGATTGGGAAAA	
si-GONAD	#2L-1	5’ intron	Intact	TTTGGGCCCCAGCTTGACCCAAGGATACAGTATCCTAGGGAAGTTACCAAAATCAGAG ATAGTATGCAGCAGCCAGGGGTCTCATGT	++
		3’ intron	Indels	AAAGATATTCAGTCTAGCCCAATGACCCCCAAGACCTCACTANACCTCCCTCCTTCAA TTTAATGATTGTGAAAAAGCCAGGAAGTACAAATGAGTGGGC	
si-GONAD	#2L-2	5’ intron	Intact	TTTGGGCCCCAGCTTGACCCAAGGATACAGTATCCTAGGGAAGTTACCAAAATCAGAG ATAGTATGCAGCAGCCAGGGGTCTCATGT	+
		3’ intron	Intact	AAAGATATTCAGTCTAGCCCAATGACCCCCAAGTACTAGACCTCACTCCTTCAGTTTA AGGATTGTGGAAAA	
si-GONAD	#2L-3	5’ intron	Intact	TTTGGGCCCCAGCTTGACCCAAGGATACAGTATCCTAGGGAAGTTACCAAAATCAGAG ATAGTATGCAGCAGCCAGGGGTCTCATGT	+
		3’ intron	Indels	AAAGATATTCAGTCTAGCCCAATGACCCCCAAGCTAGACCTCACTCCTTCAGTTTAAG GATTGTGGAAAA	
si-GONAD	#2L-4	5’ intron	Indels	TTTGGGCCCCAGCTTGACCCAAGGATACAGTATCCTAGGGAAGTTACCAAAATCAGAG ATAGTATGATGTATCTTTGATCCTCTTGGGTGGAGCT	+
		3’ intron	Indels	AAAGATATTCAGTCTAGCCCAATGACCCCCCAANTATANNACTTCNTTCCTTCNNTTAA NGGATGGGGGAAAACCCAGGAATTACAAA	
si-GONAD	#2L-5	5’ intron	Intact	TTTGGGCCCCAGCTTGACCCAAGGATACAGTATCCTAGGGAAGTTACCAAAATCAGAG ATAGTATGCAGCAGCCAGGGGTCTCATGT	+
		3’ intron	Indels	AAAGATATTCAGTCTAGCCCAATGACCCCCAAGATACTAAACCTCCCTCCTTCNNTTTA AGGATTGGGAAAAACCCAGGAAGTACAAA	
si-GONAD	#2L-6	5’ intron	Intact	TTTGGGCCCCAGCTTGACCCAAGGATACAGTATCCTAGGGAAGTTACCAAAATCAGAG ATAGTATGCAGCAGCCAGGGGTCTCATGT	-
		3’ intron	Intact	AAAGATATTCAGTCTAGCCCAATGACCCCCAAGTACTAGACCTCACTCCTTCAGTTTA AGGATTGTGGAAAA	
si-GONAD	#2L-7	5’ intron	Intact	TTTGGGCCCCAGCTTGACCCAAGGATACAGTATCCTAGGGAAGTTACCAAAATCAGAG ATAGTATGCAGCAGCCAGGGGTCTCATGT	-
		3’ intron	Intact	AAAGATATTCAGTCTAGCCCAATGACCCCCAAGTACTAGACCTCACTCCTTCAGTTTA AGGATTGTGGAAAA	
si-GONAD	#2L-8	5’ intron	Intact	TTTGGGCCCCAGCTTGACCCAAGGATACAGTATCCTAGGGAAGTTACCAAAATCAGAG ATAGTATGCAGCAGCCAGGGGTCTCATGT	+
		3’ intron	Intact	AAAGATATTCAGTCTAGCCCAATGACCCCCAAGTACTAGACCTCACTCCTTCAGTTTA AGGATTGTGGAAAA	
si-GONAD	#2R-1	5’ intron	Indels	TTTGGGCCCCAGCTTGACCCAAGGATACAGTATGCTATACGAAGTTATCCTAGGGAAG TTACCAAAATCAGAGATAGTATGCAGCAGCCAGGGGTCTCATGT	++
		3’ intron	Intact	AAAGATATTCAGTCTAGCCCAATGACCCCCAAGTACTAGACCTCACTCCTTCAGTTTA AGGATTGTGGAAAA	
simi-GONAD	#3L-6	5’ intron	Intact	TTTGGGCCCCAGCTTGACCCAAGGATACAGTATCCTAGGGAAGTTACCAAAATCAAAG ATAGTATGCAGCAGCCAGGGGTCTCATGT	ND
		3’ intron	Intact	AAAGATATTCAGTCTAGCCCAATGACCCCCAAGTACTAGACCTCACTCCTTCAGTTTA AGGATTGTGGAAAA	
simi-GONAD	#3R-5	5’ intron	Indels	TTTGGGCCCCAGCTTGACCCAAGGATACAGTTACCNNAAATNNNGAGATAGTATGCAGC AGCCAGGGGTCTCATGT	ND

		3’ intron	Intact	AAAGATATTCAGTCTAGCCCAATGACCCCCAAGTACTAGACCTCACTCCTTCAGTTTAAGGATTGTGGAAAA	
simi-GONAD	#3R-7	5’ intron	Indels	ACTATNNCTCCCAGACTTAGGGCTGNNNGATTGTCTTACCCCTCCTTGCCTCACACCCNGACAAGTCCCACGCNNNCTGTTCTCTCTGTNATGCATGACANNCA	
		3’ intron	Intact	AAAGATATTCAGTCTAGCCCAATGACCCCCAAGTACTAGACCTCACTCCTTCAGTTTAAGGATTGTGGAAAA	
simi-GONAD	#3R-8	5’ intron	Indels	GATGGGCCCCAGCTTGACCCAAGGATACAGTCTCTGCTCTGCTCAACTCCTTCATGCCTTCAAAACCAGTACCACCTGGGTGAC	ND
		3’ intron	Indels	AAAGATATTCAGTCTAGACCAATGACCTCACTCCTTCAGTTTAAGGATTGTGGAAAAGCCAGGGAGTACAGAGGAGTGGGCTTGAA	
simi-GONAD	#4L-3	5’ intron	Intact	TTTGGGCCCCAGCTTGACCCAAGGATACAGTATCCTAGGGAAGTTACCAAAATCAGAGATAGTATG	ND
		3’ intron	Intact	AAAGATATTCAGTCTAGCCCAATGACCCCCAAGTACTAGACCTCACTCCTTCAGTTTAAGGATTGTGGAAAA	

[1]*si*-GONAD was performed by the 1st *i*-GONAD with a solution containing Cas9, Mecp2-L2 gRNA, donor I ssODN and FITC-dextran, and subsequently the 2nd *i*-GONAD with a solution containing Cas9, Mecp2-R1 gRNA, and donor II ssODN. *simi*-GONAD was performed by *i*-GONAD with a solution containing Cas9, Mecp2-L2 gRNA, donor I ssODN, Mecp2-R1 gRNA and donor II ssODN.
[2]Only samples that are judged as those having ~960-bp PCR products are listed.
[3]Absence or presence of KI or indels in each of intronic site of *Mecp2* is examined by direct sequencing.
[4]Mode of KI or indels at 5’ and 3’ intronic region interposing exon 3 of murine *Mecp2* is judged by direct sequencing of PCR products and expressed as KI, indels or intact. The samples showing lack of relatively large-sized region are included as those with indels. The samples showing complexed pattern of ideograms suggest the presence of a mixture of several mutations in one embryo. Thus, these samples are classified as “Indels”. ND, not determined due to the lack of ~960-bp PCR products.
[5]Sequences of 5’ or 3’ intronic region of *Mecp2,* in which the site recognized by Mecp2-L2 gRNA or Mecp2-R1 gRNA is included. [6]Intensity of fluorescence in each embryo after observation under a fluorescence microscope is determined as – (no fluorescence), ± (very fain fluorescence), + (moderate fluorescence), and ++ (strong fluorescence). ND, not determined.
